# Comprehensive Investigation of Machine Learning and Deep Learning Networks for Identifying Multispecies Tomato Insect Images

**DOI:** 10.3390/s24237858

**Published:** 2024-12-09

**Authors:** Chittathuru Himala Praharsha, Alwin Poulose, Chetan Badgujar

**Affiliations:** 1 School of Data Science, Indian Institute of Science Education and Research Thiruvananthapuram (IISER TVM), Vithura, Thiruvananthapuram 695551, India; chimalapraharsha21@iisertvm.ac.in; 2 Biosystems Engineering and Soil Sciences, The University of Tennessee, Knoxville, TN 37996, USA

**Keywords:** smart agriculture, pest detection systems, pest monitoring, integrated pest management, convolution neural network, optimizers, machine learning, deep learning

## Abstract

Deep learning applications in agriculture are advancing rapidly, leveraging data-driven learning models to enhance crop yield and nutrition. Tomato (*Solanum lycopersicum*), a vegetable crop, frequently suffers from pest damage and drought, leading to reduced yields and financial losses to farmers. Accurate detection and classification of tomato pests are the primary steps of integrated pest management practices, which are crucial for sustainable agriculture. This paper explores using Convolutional Neural Networks (CNNs) to classify tomato pest images automatically. Specifically, we investigate the impact of various optimizers on classification performance, including AdaDelta, AdaGrad, Adam, RMSprop, Stochastic Gradient Descent (SGD), and Nadam. A diverse dataset comprising 4263 images of eight common tomato pests was used to train and evaluate a customized CNN model. Extensive experiments were conducted to compare the performance of different optimizers in terms of classification accuracy, convergence speed, and robustness. RMSprop achieved the highest validation accuracy of 89.09%, a precision of 88%, recall of 85%, and F1 score of 86% among the optimizers, outperforming other optimizer-based CNN architectures. Additionally, conventional machine learning models such as logistic regression, random forest, naive Bayes classifier, support vector machine, decision tree classifier, and K-nearest neighbors (KNN) were applied to the tomato pest dataset. The best optimizer-based CNN architecture results were compared with these machine learning models. Furthermore, we evaluated the cross-validation results of various optimizers for tomato pest classification. The cross-validation results demonstrate that the Nadam optimizer with CNN outperformed the other optimizer-based approaches and achieved a mean accuracy of 79.12% and F1 score of 78.92%, which is 14.48% higher than the RMSprop optimizer-based approach. The state-of-the-art deep learning models such as LeNet, AlexNet, Xception, Inception, ResNet, and MobileNet were compared with the CNN-optimized approaches and validated the significance of our RMSprop and Nadam-optimized CNN approaches. Our findings provide insights into the effectiveness of each optimizer for tomato pest classification tasks, offering valuable guidance for practitioners and researchers in agricultural image analysis. This research contributes to advancing automated pest detection systems, ultimately aiding in early pest identification and proactive pest management strategies in tomato cultivation.

## 1. Introduction

Tomato is the most produced vegetable in the world, with a production of 189 million tons in 2021 [1]. Fresh, processed, or tomato-based products are common in the human diet and cuisines in multiple countries and provide essential food nutrients. For example, tomato is the second most consumed vegetable in the U.S., with a per capita availability of 14.3 kg/per person [2]. Global tomato consumption is expected to increase due to increased processing needs, diet shifts, consumer preferences, and increased importance of food nutrition and a balanced diet [3,4,5]. Therefore, tomato cultivation is a critical component of the global food supply chain to meet the food and nutrition demand of growing global populations [6]. However, tomato crops are vulnerable to various pests and diseases, which can significantly reduce yields and fruit quality if not promptly managed [7,8]. Insect pests can attack tomato crops from early growth to late-maturity stages, targeting plant leaves, flowers, and fruits. This leads to crop damage, reduced yield, or low-quality fruits, affecting overall production and farmers’ income [9], further putting human food nutrition at risk.

Integrated pest management (IPM) is the most commonly used and holistic approach to managing insect pests [10,11]. The IPM program employs a combination of techniques to prevent pests from becoming a threat. Monitoring crops for pest infestation and attack is the primary first step of the IPM program, and its success largely depends on early insect-pest detection and identification [12]. Traditional pest detection and classification methods often rely on manual inspection, which can be time-consuming, labor-intensive, and prone to human error. Also, accurate insect-pest species identification requires expertise or domain knowledge and inaccurate identification can lead to a waste of resources, effort, and money. Pest control measures do vary with species type, and different species carry or transmit plant viruses. Therefore, detecting different pests in large fields is often challenging. In recent years, computer vision and deep learning (DL) advances have enabled the development of automated pest recognition systems using image analysis techniques [13,14,15,16], which can be easily scaled and adapted to a robotic system for automated pest recognition tasks [17]. Machine learning (ML) models are utilized to identify and categorize pests, aiding in reducing labor costs and the targeted chemical application [18,19]. Smart or precision agriculture, using intelligent technology and pest recognition research, is expanding quickly [20]. Convolutional Neural Networks (CNNs), particularly, have shown remarkable success in image classification tasks, including identifying agricultural pests and diseases [21,22,23,24]. By leveraging large, annotated image datasets, CNNs can learn discriminative features and patterns, allowing for accurate and efficient pest classification. Pest recognition detects and classifies pests on crops, which helps farmers take appropriate action to prevent financial and food loss.

In this study, our research focuses on classifying the most common tomato pests using CNN models. The objective is to develop a robust and automated system that accurately identifies common tomato pests based on visual cues captured in images. This research addresses the pressing need for effective IPM strategies in tomato cultivation, aiming to mitigate crop losses and enhance agricultural productivity. The tomato pest recognition task presents several challenges, including the diverse appearance of pests at different life stages, variations in lighting and environmental conditions, and the presence of occlusions and clutter in images. Additionally, selecting an appropriate optimizer for training CNN models is crucial for achieving optimal classification performance. Optimizers play a critical role in updating model parameters during training, influencing factors such as convergence speed, generalization ability, and robustness. Despite this, optimizers are often selected based on either the modeler’s experience or random/limited evaluation, which often lacks detailed investigation. To address these challenges, we conducted a comprehensive investigation into the impact of various optimizers on the classification of tomato pest images using CNNs. By systematically evaluating different optimizers, including AdaDelta [25], AdaGrad [26], Adam [27], RMSprop, Stochastic Gradient Descent (SGD), and Nadam [28,29], we aim to identify the most effective approach for tomato pest classification tasks. Our research contributes to advancing automated pest recognition systems in agriculture, facilitating early pest identification and proactive pest management strategies to ensure the production and productivity of tomato crops. The significant contributions of our study are summarized below:

1. Development of a Tomato Pest Classification System Using CNNs: We designed and implemented a CNN-based classification framework tailored to identify eight common tomato pests: Spider mites (*Tetranychus urticae*—TU), Whitefly (*Bemisia argentifolii*—BA), Melon fruit fly (*Zeugodacus cucurbitae*—ZA), Melon thrips (*Thrips palmi*—TP), Green peach aphid (*Myzus persicae*—MP), Tobacco cutworm (*Spodoptera litura*—SL), Beet armyworm (*Spodoptera exigua*—SE), and Fruit borer (*Helicoverpa armigera*—HA). Our system evaluates CNN performance using accuracy, precision, recall, F1 score, the area under the curve (AUC), receiver operating characteristic (ROC) curves, and confusion matrices.
2. Comprehensive Optimizer Evaluation: We systematically analyzed the effect of six optimizers on CNN performance, providing actionable insights into their convergence properties and classification efficacy. Our results demonstrated that RMSprop emerged as the best-performing optimizer in traditional training/validation splits, while Nadam showed superior performance in cross-validation scenarios.

Our findings significantly advance automated pest detection systems in agriculture, providing an innovative, practical solution to mitigate crop losses and enhance productivity. This research not only underscores the critical role of optimizers in CNN training but also establishes a benchmark for tomato pest classification, offering actionable insights and paving the way for future developments in agricultural technology. The rest of the paper is organized as follows: Section 2 provides an overview of related work on tomato pest classification and recent research trends. Section 3 presents a system overview of the pest classification system using the CNN model. Section 4 discusses the experiment and result, and Section 5 addresses the challenges and limitations of the tomato pests classification system. Finally, Section 6 concludes this paper with future research directions.

## 2. Related Work

The classification of tomato pests through image analysis techniques has recently attracted significant attention, fueled by the growing need for automated pest detection and management in agriculture [30]. Researchers have explored a variety of approaches and methodologies to address this challenge, capitalizing on advancements in computer vision [31], machine learning [32], and deep learning [33,34,35,36,37]. In particular, recent developments in these technologies have enabled their application to real-world scenarios, including agriculture, where they play a critical role in addressing complex tasks [38,39,40]. This study reviews the current research trends and progress in tomato pest detection and classification, highlighting the contributions of machine and deep learning models in advancing this domain.

Ref. [41] proposed a machine learning-based approach that used feature extraction methods such as local binary patterns (LBP), gray level co-occurrence matrix (GLCM), histogram of oriented gradient (HOG), and speeded up robust features (SURF) to extract textural information from pest images. The outcomes of their experiment demonstrated how well the SVM classifier performed in classifying tomato pest images based on the feature that the local binary patterns (LBP) technique extracted. A pre-trained deep learning method for tomato crop disease classification was proposed by [42]. The PlantVillage dataset’s images of tomato leaves with diseases and healthy conditions were utilized by the authors as the input for two deep learning-based networks (i.e., AlexNet and VGG16). The model effectiveness was assessed by varying the number of images, minibatch sizes, weight, and bias learning rate. The authors concluded that there was no discernible relationship between classification accuracy and the AlexNet minibatch size adjustment. However, as the minibatch size increased, the accuracy declined in the VGG16. An enhanced deep residual network method was proposed for tomato pest diagnosis [43], which accurately identified the tomato pests, including melon flies, cotton borers, green peach aphids, beet armyworms, taro insects, whiteflies, and spider mites. Ref. [44] developed an algorithm for real-time identification of pests and tomato diseases in their natural environment. Better detection performance was demonstrated when the proposed approach was used in real-world tomato fields. The experimental findings suggested that the proposed approach enhanced the ability to detect small and occluded objects. A deep learning framework for tomato pest recognition was proposed by [45]. The approach leveraged pre-trained models and modified them to classify tomato pests by combining CNNs with transfer learning techniques, which resulted in good disease and pest identification outcomes. Ref. [46] proposed a lightweight, multi-scale tomato pest and disease categorization network (CNNA). The authors suggested a multi-scale feature fusion module to enhance the model’s capacity to extract features for various pests and spot sizes. Additionally, they offered a global channel attention technique to increase the model’s sensitivity to detect pest traits. According to the experimental findings, the pre-trained lightweight models, including MobileNetV3, MobileVit, and ShuffleNetV2, performed worse in classification accuracy than the proposed CNNA model. A learning algorithm for autonomous scouting robots using internal databases for tomato pest detection and identification was introduced by [47]. They developed a dataset comprising images of infected tomato plants to develop and assess machine and deep learning models, which resulted in a reliable and effective approach to identify pests on tomato plants. Ref. [48] refined filter bank framework for identifying pests and diseases in tomato plants to address the issues of class imbalance and false positives. The suggested method can handle class imbalances and false positives produced by the bounding box generator by implementing a refinement filter bank framework, especially for sparse data. Ref. [49] presented a novel technique for employing a lightweight network to identify tomato diseases and pests. Their investigation looked at the effects of dataset balance, data amount, and hyperparameters to improve model performance for diagnosing tomato diseases and pests. The outcomes of their experiments demonstrated the benefits of their suggested paradigm for tasks involving the classification of tomato pests. A real-time DL-based detector for identifying tomato diseases and pests was proposed by [50]. The study compared the performance of the Single-Shot Multibox Detector (SSD), Region-based Fully Convolutional Network (R-FCN), and Faster Region-based Convolutional Neural Network (Faster R-CNN) on a benchmark dataset of tomato images, including nine distinct pests and disease types, along with intra- and inter-class differences. The study compared the advantages and disadvantages of each model for the pest classification task. Furthermore, they discovered superior classification performance when data augmentation was used. Ref. [51] proposed an improved YOLOv3 network for tomato disease and pest identification based on a DL-based object detection method. The authors created a dataset of pests and diseases that affect tomatoes in their actual natural habitat. The authors increase the YOLOv3 model’s detection accuracy and speed by using an image pyramid to accomplish multi-scale feature detection in the feature layer. Their findings suggest that improved YOLOv3 outperforms conventional detection methods in terms of performance.

The availability of large, annotated datasets, including images of tomato pests, has dramatically accelerated the progress of model building and benchmarking in the domain. These datasets have been carefully selected to cover a wide range of tomato pest species, allowing a comprehensive analysis and comparison of various methods. These datasets offer a rich tapestry of annotated images showing different phases, morphologies, and pest infestation levels, which are priceless resources. These datasets’ comprehensiveness creates an ideal atmosphere for carefully analyzing and contrasting various methods. A large dataset of tomato leaves is generated by the authors of [52], which is again split into two datasets based on image sources. In the first dataset, tomato leaf images are taken from the PlantVillage database and are divided into ten categories (one for healthy and nine for diseases). In [53], a multispectral dataset is suggested to identify two pests, i.e., *tuta absoluta* and *leveillula taurica*, on tomato plants. This dataset contains a total of 263 multispectral images taken in a greenhouse. The authors also used the baseline Faster-RCNN object detector to locate and categorize lesions. Ref. [54] presents another well-known tomato disease dataset, which includes more than fifty thousand images of different crops such as tomatoes, potatoes, grapes, apples, corn, blueberries, raspberries, soybeans, squash, and strawberries. Researchers can use various machine learning and computer vision techniques to address the difficulties in tomato pest recognition tasks, from conventional approaches to cutting-edge deep learning techniques [55,56]. Additionally, annotated dataset availability makes benchmarking easier and enables researchers to evaluate algorithm performance against established criteria objectively. Researchers can ultimately drive innovation and advancement in the field by identifying areas of strength, weakness, and improvement in their algorithms with standardized datasets. Large-scale, annotated databases of tomato pests are essential for model-building and benchmarking improvements. These datasets give researchers the tools to create, hone, and assess algorithms to solve the most critical problems in agricultural pest recognition.

Integrating many data sources, including spectral data, visual images, and environmental variables, is a novel strategy for improving the precision and resilience of tomato pest identification systems [57,58]. Researchers hope to enhance the efficacy of classification algorithms by capturing more comprehensive images of the pest-infested tomato crop environment by combining data from many modalities. The primary source of information is visual images, which offer comprehensive visual representations of tomato plants and related insect infestations [59]. The morphology, size, color, and geographic distribution of pests on tomato leaves, stems, and fruits are all visible in these images. Spectral data from remote sensing technologies, including hyperspectral or multispectral imaging, provide additional information about tomato plants’ physiological state and metabolic makeup in addition to visual data [60]. It may not be possible to discern minute differences in plant health, stress levels, and pest-induced damage from visual images alone, but spectral signatures recorded at various wavelengths can [61]. In addition, environmental factors that affect crop sensitivity, pest population dynamics, and pest activity include temperature, humidity, soil moisture, and light intensity. Researchers can consider the larger ecological context in which pest infestations occur by incorporating environmental data into classification models, which increases the model’s predicted accuracy and resilience [62,63]. By integrating data from several modalities, researchers can take advantage of the complementing information each source offers. For instance, spectral data may show minute modifications in plant physiology that point to pest stress. At the same time, visual images offer precise spatial details regarding the distribution of pests and the extent of their damage. Several technological difficulties arise when integrating data from many modalities, such as data fusion, feature extraction, and model integration. Scholars utilize sophisticated data fusion methodologies, including feature-level fusion, decision-level fusion, or model-level fusion, to efficiently merge data from several sources while maintaining the unique attributes of each modality. Integrating various data sources presents a viable way to improve tomato pest detection and classification systems’ robustness and accuracy. Using spectral data, visual pictures, and environmental variables, which offer complementary information, researchers can create more comprehensive and valuable models for managing pests in tomato crops [64].

Researchers have been delving further into advanced image processing techniques to improve the quality and relevance of input data for classification models and increase the effectiveness of tomato pest recognition systems. Some image processing techniques for automated leaf pest and disease recognition are reported in [30,65,66,67,68]. These sophisticated techniques include object detection, segmentation, and feature extraction, all of which help to reduce image noise and isolate relevant characteristics. Together, these cutting-edge image-processing methods help improve the accuracy and applicability of tomato pest classification models. Separating pertinent features, decreasing image noise, and enabling accurate localization of areas contaminated by pests allow classification models to identify minute indicators of pest presence with increased precision and dependability. As a result, researchers can develop more effective and reliable systems for tomato pest detection and classification, thereby advancing agricultural sustainability and crop protection efforts.

When examining pest infestations in tomato fields, field surveys and monitoring systems with cameras and sensors are essential tools [69,70]. These technologies facilitate real-time data collection, analysis, and decision-making, empowering farmers and stakeholders to implement proactive IPM programs. Early detection and proactive management of tomato pests are made possible by field surveys and monitoring systems fitted with cameras and sensors [71]. In the end, these systems contribute to resilient and sustainable agricultural systems.

This extensive literature review summarizes the most recent methods for automatically identifying and categorizing pests and diseases that affect tomato plants. The authors underlined the significance of precise pest identification for efficient crop management, including conventional image processing techniques and machine learning approaches. By showcasing the promise of deep learning techniques for precise and automated pest detection in agriculture, the works reported here advance the field of tomato pest classification utilizing image analysis methods. Our study intends to investigate further optimizers’ impact on the classification of tomato pest images by expanding on previous research and approaches. This will provide essential insights for enhancing model performance and valuable applications in crop management.

## 3. System Overview of Tomato Pest Classification

**Figure 1:**
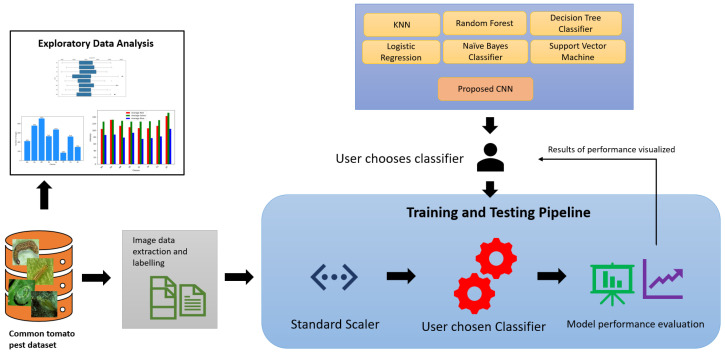
System overview of tomato pest classification using machine and deep learning models.

Classifying tomato pests using images involves integrating various components, including data collection, pre-processing, model development, training, and evaluation. Figure 1 shows the overview of tomato pest classification using the machine and deep learning approach. By following the system overview of tomato pest classification, researchers and practitioners can develop a robust and automated solution for classifying tomato pests using images.

### 3.1. Data Collection

**Figure 2:**
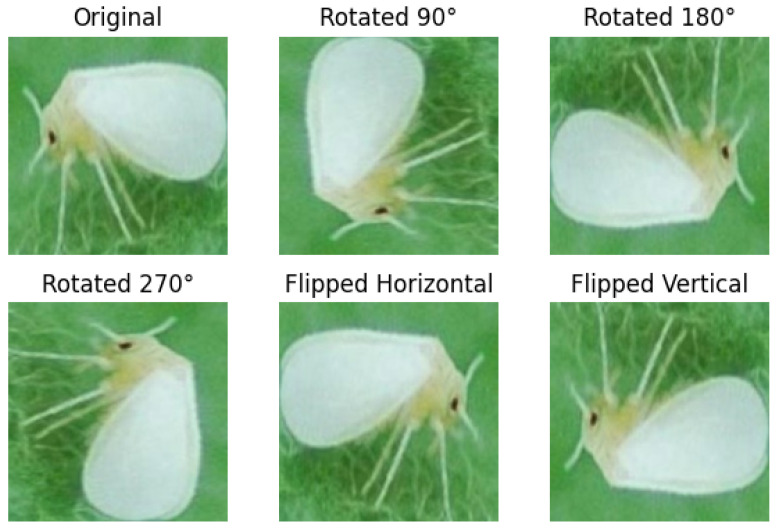
Examples of data augmentation used in the tomato pest image dataset.

The system begins by collecting a diverse dataset of tomato pest images. We used a database of eight common tomato crop pests developed by the authors of [72]. The dataset included eight pest classes, which are as follows: (1) Spider mites (*Tetranychus urticae*)—TU, (2) Whitefly (*Bemisia argentifolii*)—BA, (3) Melon fruit fly (*Zeugodacus cucurbitae*)—ZA, (4) Melon trhips (*Thrips palmi*)—TP, (5) Green peach aphid (*Myzus persicae*)—MP, (6) Tobacco cutworm (*Spodoptera litura*)—SL, (7) Beet armyworm (*Spodoptera exigua*)—SE, and (8) Fruit borer (*Helicoverpa armigera*) HA. The dataset is annotated with labels of specific pests in each image. The image repository comprises 609 original images categorized into eight distinct groups. The database was augmented by applying image enhancement techniques, such as rotation (90°, 180°, 270°), flip (horizontal and vertical), and cropping, resulting in 4263 images (Figure 2). Standardization procedures were employed to unify the dimensions of all images to 299 × 299 pixels, with the file standardized to JPG format. This database serves as a comprehensive collection of visual data, aiding researchers and practitioners in studying and understanding various tomato pest species [73,74,75,76].

### 3.2. Data Preprocessing

The selected dataset undergoes an extensive preprocessing pipeline to ensure it is well prepared for model training and capable of yielding accurate classification results. This preprocessing involves several essential tasks, such as resizing images to a uniform size, normalizing pixel values, employing augmentation techniques, and encoding labels into numerical formats. These steps aim to enhance dataset consistency, improve model generalization, and optimize computational efficiency. In the image resizing stage, all tomato pest images are resized to a fixed resolution (297 × 297 pixels) to maintain uniformity across the dataset. This step ensures that images of varying dimensions are standardized, reducing computational complexity during training while preserving essential features required for accurate pest classification. Resizing also facilitates batch processing, enabling the model to process data more efficiently. Normalization is applied to the pixel values of tomato pest images, scaling them to a standard range, typically between 0 and 1. This technique minimizes the impact of pixel value disparities between images, promoting faster convergence during training and improving the stability of the optimization process. Normalization mitigates the risk of numerical instability and enhances the model’s ability to learn effectively by ensuring that all input data shares a similar scale. Data augmentation is another critical step in preprocessing, aimed at increasing dataset diversity and improving the model’s generalization capabilities. The system generates additional training samples from existing images by applying rotation, flipping, cropping, scaling, and brightness adjustments. These augmented images simulate real-world variations, such as changes in orientation, lighting conditions, or partial occlusions, enabling the model to become more robust when deployed. This approach expands the dataset size and helps prevent overfitting, ensuring that the model performs well on unseen data. Finally, label encoding is employed to convert categorical labels of tomato pests into numerical representations suitable for deep learning models. For example, each pest class is assigned a unique integer value. This step ensures compatibility with classification algorithms, which require numerical inputs to compute loss and adjust weights during training. Furthermore, this encoding simplifies the processing of class labels and facilitates multi-class classification tasks, where the model distinguishes between numerous pest species. These preprocessing steps create a robust and reliable foundation for training the machine and deep learning models. By addressing challenges such as varying image dimensions, diverse pixel value ranges, limited dataset size, and categorical labels, the preprocessing pipeline ensures that the dataset is comprehensive and optimized for achieving high-performance tomato pest classification.

### 3.3. Model Development and Training

**Figure 3:**
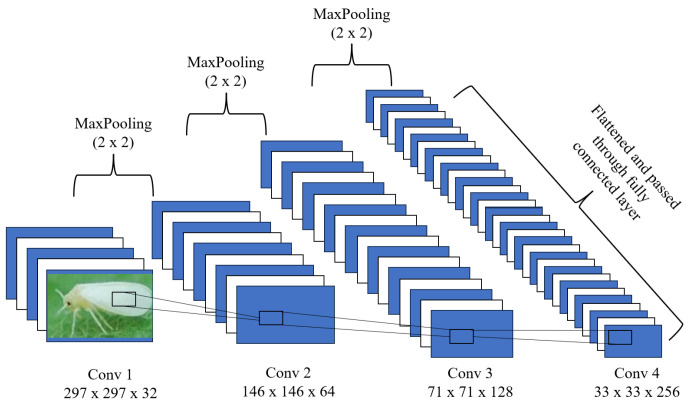
CNN model used for the optimizer performance analysis on tomato pest image dataset.

Developing a CNN architecture is pivotal in achieving high accuracy for tomato pest image classification. This tailored CNN architecture is designed to handle the complexities of image classification tasks in agricultural applications. Tomato pest image classification using CNN represents a significant advancement in leveraging artificial intelligence to address agricultural challenges. By automatically learning and extracting intricate features from images, CNNs have revolutionized the identification and categorization of pests, enabling precise pest detection and management strategies. Training the CNN on diverse datasets of tomato pest images ensures that the model can accurately distinguish between pest species and their developmental stages. This capability is critical for early pest detection and precise intervention, mitigating crop damage and improving agricultural productivity. By analyzing unique patterns, textures, and shapes present in pest images, the CNN effectively automates the previously labor-intensive and error-prone task of pest identification. Figure 3 illustrates the architecture of the proposed CNN model employed for tomato pest image classification. The CNN operates by computing feature maps for each layer and sequentially passing them to subsequent layers in a process known as forward propagation. At the end of the forward pass, the model predicts outputs, and the loss is calculated based on the deviation of predictions from the true labels. The backpropagation algorithm is then utilized to minimize the loss by iteratively updating the model’s weights through gradient descent. This iterative optimization allows CNNs to improve their predictions progressively during training. The CNN architecture consists of a single convolutional layer, which is instrumental in extracting basic visual features, such as edges and textures, from the input images. This layer is followed by a batch normalization layer, designed to stabilize the training process and mitigate the problem of co-variate shift. By normalizing the inputs to each layer, batch normalization enhances the convergence speed and overall performance of the model. To further refine the extracted features, a max-pooling layer is incorporated, which reduces the spatial dimensions of the feature maps while retaining the most salient features. This step also reduces noise, ensuring irrelevant image details do not adversely affect model performance. The processed feature maps are then passed into dense layers, which perform high-level reasoning and classification tasks. To prevent overfitting, a dropout strategy is employed within the dense layers. Dropout randomly deactivates a fraction of neurons during training, forcing the network to learn more robust and generalized features. This approach ensures that the model does not merely memorize the training data but develops the ability to generalize effectively to unseen images. By integrating these components, the CNN architecture is tailored to achieve high accuracy and robustness in classifying tomato pest images. Combining convolutional layers, normalization, pooling, dense layers, and regularization techniques ensures the model can handle the diverse and complex challenges of agricultural image data. This system forms a foundation for advancing automated pest detection tools, contributing to sustainable and efficient pest management practices.

The first convolutional layer processes the input image of size 297 × 297 × 1 using 32 filters, each with a size of 3 × 3 with 320 trainable parameters (Table 1). This is followed by a max-pooling layer with a kernel size of 2 × 2 (Figure 3). The second convolutional layer employs 64 filters of size 3 × 3 with 18,496 trainable parameters (Table 1), followed by another max-pooling layer. The third and fourth convolutional layers utilize 128 and 256 size 3 × 3 filters, respectively having 295,168 trainable parameters. Each convolutional layer uses the Rectified Linear Unit (ReLU) activation function to prevent the vanishing gradients problem during backpropagation. The ReLU function is defined as:

(1) ReLU(x)=max(0,x)

ReLU outputs *x* if *x* is positive, and zero otherwise.

The features extracted from the convolutional layers are flattened and then passed through two fully connected dense layers containing 512 and 214 units, respectively (Figure 3). Dropout layers follow these dense layers to regularize the model and enhance its performance. The output layer generates class probabilities using the SoftMax activation function, defined as:

(2) $$\mathrm{SoftMax}\left(x_{i}\right)=\frac{e^{x_{i}}}{\sum_{j=1}^{n}e^{x_{j}}}$$

where $x_{i}$ is the *i*-th element of the input vector *x* and $\sum_{j=1}^{n}e^{x_{j}}$ is the sum of the exponential functions applied to all elements $x_{j}$ of the input vector *x*, where *j* ranges from 1 to *n*, and *n* is the total number of elements in *x* which is eight in our case.

The max-pooling layer, which reduces noise, feeds the feature maps into the dense layers. A dropout strategy is employed to prevent overfitting. Data augmentation techniques such as a rotation range of 20 degrees, shearing of 20%, zooming of 20%, and horizontal flipping are used to increase the dataset’s diversity.

The prepared dataset is divided into training, validation, and test subsets. The CNN model is trained on a training subset, where it learns to extract relevant features from the input images and classify them into respective pest categories. During training, an optimizer is employed to update the model parameters iteratively, minimizing a defined loss function.

### 3.4. Optimizers

The choice of optimizer plays a crucial role in training the CNN model. Standard optimizers, including AdaDelta, AdaGrad, Adam, RMSprop, SGD, and Nadam, are used in this study to analyze the classification performance of tomato pest images. Each optimizer has its strengths and weaknesses in terms of convergence speed, stability, and robustness to hyperparameters. The system experiments with different optimizers to identify the most suitable one for the tomato pest classification task. The impact of these optimizers on the classification of tomato pest images via CNNs can vary based on factors such as dataset size, model architecture, and hyperparameter settings. Through empirical evaluation and comparison, researchers can identify the optimizer that best balances convergence speed, classification accuracy, and robustness for this specific task. This knowledge can inform the development of more efficient and effective DL models for pest recognition.

#### 3.4.1. AdaDelta

The AdaDelta optimizer is an adaptive learning rate optimization algorithm designed to address some of the limitations of other adaptive learning rate methods like AdaGrad and RMSProp. AdaDelta offers a robust and efficient optimization algorithm for training neural networks. It is beneficial when dealing with large-scale datasets or complex models where manual tuning of learning rates may be challenging. In practice, AdaDelta is often used alongside other popular optimizers like Adam and RMSProp, providing researchers and practitioners with a versatile tool for training DL models. Algorithm 1 summarizes the AdaDelta optimizer used in our tomato pest image classification.
**Algorithm 1** AdaDelta Optimizer**Input:** Initial parameters θ, decay rate ρ, small constant ϵ **Output:** Updated parameters θ Initialize accumulated gradients $E[\Delta \theta^{2}]=0$, accumulated updates E[Δθ]=0 **While** not converged **do**:Compute gradient *g* on mini-batch.Update the accumulated squared gradients:
$E\left[\Delta \theta^{2}\right]\leftarrow \rho \cdot E\left[\Delta \theta^{2}\right]+\left(1-\rho \right)\cdot g^{2}$Compute the root mean square (RMS) value of the parameter updates:
$\mathrm{rms}\left(\Delta \theta \right)\leftarrow \sqrt{E[\Delta \theta^{2}]+\epsilon }$Compute the parameter update:
$\Delta \theta \leftarrow -\frac{\mathrm{rms}(\Delta \theta )}{\mathrm{rms}(\Delta \theta )+\epsilon }\cdot g$Update the accumulated updates:
$E\left[\Delta \theta \right]\leftarrow \rho \cdot E\left[\Delta \theta \right]+\left(1-\rho \right)\cdot \Delta \theta^{2}$Update the parameters using the computed update:
θ←θ+Δθ


#### 3.4.2. AdaGrad (Adaptive Gradient Algorithm)

AdaGrad adapts the learning rate for each parameter based on the historical gradients for that parameter. It performs well in settings with sparse data because it effectively reduces the learning rate for parameters that have received frequent updates. However, AdaGrad may suffer from aggressively diminishing learning rates, leading to premature convergence and slow progress in training. Algorithm 2 summarizes the AdaGrad optimizer process used in the tomato pest image classification.
**Algorithm 2** AdaGrad Optimizer**Input:** Initial parameters θ, small constant ϵ **Output:** Updated parameters θ Initialize accumulated squared gradients G=0 **While** not converged **do**:Compute gradient *g* on mini-batch.Update the accumulated squared gradients:
$G\leftarrow G+g^{2}$Compute the root mean square (RMS) value of the accumulated squared gradients:
$\mathrm{rms}\left(G\right)\leftarrow \sqrt{G+\epsilon }$Compute the parameter update:
$\Delta \theta \leftarrow -\frac{g}{\mathrm{rms}(G)}$Update the parameters using the computed update:
θ←θ+Δθ


#### 3.4.3. Stochastic Gradient Descent (SGD)

SGD is a fundamental optimizer commonly used to train neural networks. It updates model parameters based on the gradient of the loss function with respect to the parameters. While SGD is simple and computationally efficient, it may suffer from slow convergence and sensitivity to learning rate tuning. Algorithm 3 shows how the SGD optimizer works for tomato pest image classification.
**Algorithm 3** Stochastic Gradient Descent (SGD)**Input:** Initial parameters θ, learning rate α **Output:** Updated parameters θ **While** not converged **do**:Sample a mini-batch from the training data.Compute gradient *g* on the mini-batch.Compute parameter update:
Δθ←−α·gUpdate parameters using the computed update:
θ←θ+Δθ


#### 3.4.4. Adam (Adaptive Moment Estimation)

Adam is an adaptive learning rate optimization algorithm that computes adaptive learning rates for each parameter. It combines the advantages of both AdaGrad and RMSProp, making it well suited for a wide range of deep learning tasks. Adam often converges faster than traditional optimizers like SGD and is less sensitive to learning rate tuning. The working of the Adam optimizer is defined in Algorithm 4.
**Algorithm 4** Adam Optimizer**Input:** Initial parameters θ, learning rate α, decay rates $\beta_{1},\beta_{2}$, small constant ϵ **Output:** Updated parameters θ Initialize time step t=0, first moment estimate $m_{0}=0$, second moment estimate $v_{0}=0$ **While** not converged **do**:Increment time step: t←t+1Compute gradient *g* on mini-batch.Update first moment estimate:
$m_{t}\leftarrow \beta_{1}\cdot m_{t-1}+\left(1-\beta_{1}\right)\cdot g$Update second moment estimate:
$v_{t}\leftarrow \beta_{2}\cdot v_{t-1}+\left(1-\beta_{2}\right)\cdot g^{2}$Correct bias in first moment estimate:
${\hat{m}}_{t}\leftarrow \frac{m_{t}}{1-\beta_{1}^{t}}$Correct bias in second moment estimate:
${\hat{v}}_{t}\leftarrow \frac{v_{t}}{1-\beta_{2}^{t}}$Compute parameter update:
$\Delta \theta \leftarrow -\alpha \cdot \frac{{\hat{m}}_{t}}{\sqrt{{\hat{v}}_{t}}+\epsilon }$Update parameters using the computed update:
θ←θ+Δθ


#### 3.4.5. RMSprop (Root Mean Square Propagation)

RMSprop is another adaptive learning rate optimization algorithm that divides the learning rate for a parameter by the root mean square of recent gradients for that parameter. It addresses the rapidly diminishing learning rates in AdaGrad by using a moving average of squared gradients. RMSprop can effectively train deep neural networks, mainly when dealing with sparse data or non-stationary objectives. The RMSprop optimizer used in the tomato pest image classification is presented in Algorithm 5.

#### 3.4.6. Nadam Optimizer

The Nadam optimizer, short for Nesterov-accelerated Adaptive Moment Estimation, is a variant of the Adam optimizer that combines Nesterov momentum with Adam’s adaptive learning rate features. The Nadam optimizer addresses some limitations of Adam’s and Nesterov’s momentum, offering improved convergence speed and performance, particularly in deep neural network training. The Nadam optimizer used in the tomato pest image classification is summarized in Algorithm 6.
**Algorithm 5** RMSprop Optimizer**Input:** Initial parameters θ, learning rate α, decay rate ρ, small constant ϵ **Output:** Updated parameters θ Initialize accumulated squared gradients $E[g^{2}]=0$ **While** not converged **do**:Compute gradient *g* on mini-batch.Update accumulated squared gradients:
$E\left[g^{2}\right]\leftarrow \rho \cdot E\left[g^{2}\right]+\left(1-\rho \right)\cdot g^{2}$Compute the root mean square (RMS) value of the accumulated squared gradients:
$\mathrm{rms}\left(E\left[g^{2}\right]\right)\leftarrow \sqrt{E[g^{2}]+\epsilon }$Compute parameter update:
$\Delta \theta \leftarrow -\frac{\alpha \cdot g}{\mathrm{rms}(E\left[g^{2}\right])}$Update parameters using the computed update:
θ←θ+Δθ


**Algorithm 6** Nadam Optimizer**Input:** Initial parameters θ, learning rate α, decay rates $\beta_{1},\beta_{2}$, small constant ϵ **Output:** Updated parameters θ Initialize time step t=0, first moment estimate $m_{0}=0$, second moment estimate $v_{0}=0$ **While** not converged **do**:Increment time step: t←t+1Compute gradient *g* on mini-batch.Update first-moment estimate with Nesterov acceleration:
$m_{t}\leftarrow \beta_{1}\cdot m_{t-1}+\left(1-\beta_{1}\right)\cdot g$Update second moment estimate:
$v_{t}\leftarrow \beta_{2}\cdot v_{t-1}+\left(1-\beta_{2}\right)\cdot g^{2}$Correct bias in first moment estimate:
${\hat{m}}_{t}\leftarrow \frac{m_{t}}{1-\beta_{1}^{t}}$Correct bias in second moment estimate:
${\hat{v}}_{t}\leftarrow \frac{v_{t}}{1-\beta_{2}^{t}}$Compute parameter update:
$\Delta \theta \leftarrow -\alpha \cdot \frac{{\hat{m}}_{t}}{\sqrt{{\hat{v}}_{t}}+\epsilon }$Update parameters using the computed update:
θ←θ+Δθ


### 3.5. Evaluation

After training, the performance of the CNN model is evaluated using the validation set. Evaluation metrics such as classification accuracy, precision, recall, F1 score, confusion matrix, receiver operating characteristic (ROC) curve, and area under the curve (AUC) are computed to assess the model’s ability to classify tomato pest images correctly. These evaluation metrics help researchers and practitioners evaluate the performance of tomato pest image classification models and identify areas for improvement. By considering multiple metrics, stakeholders can comprehensively understand the model’s strengths and weaknesses and make informed decisions to enhance its performance. The performance of different optimizers is compared based on these metrics to determine their impact on classification accuracy and convergence.

Accuracy: It measures the proportion of correctly classified tomato pest images from the dataset’s total number of tomato pest images. It provides an overall assessment of the model’s performance but may not be suitable for imbalanced datasets. The accuracy of tomato pest image classification is defined as:

(3) $$\mathrm{Accuracy}=\frac{TP+FP}{TN+TP+FP+FN}$$

where *TP* stands for true positive, *FP* is false positive, *TN* is the true negative, and *FN* means false negative.

Loss Function: The loss function used is sparse categorical cross-entropy loss for both model training and validation, which is defined as:

(4) $$\left(y,\hat{y}\right)=-\sum_{i}y_{i}.\log\left({\hat{y}}_{i}\right)$$

where *y* is the true distribution, $\hat{y}$ is the predicted distribution, $y_{i}$ and ${\hat{y}}_{i}$ are the elements of the true and predicted distributions.

Precision: It measures the proportion of true positive predictions (correctly classified pest images) out of all positive predictions (both true positives and false positives). Precision indicates the model’s ability to avoid misclassifying non-pest images as pest images.

(5) $$P=\frac{TP}{TP+FP}$$

Recall (Sensitivity): It measures the proportion of true positive predictions out of all actual positive instances (both true positives and false negatives). Recall indicates the model’s ability to capture all instances of pest images in the dataset.

(6) $$R=\frac{TP}{TP+FN}$$

F1 Score: It is the harmonic mean of precision and recall. It provides a balanced measure of the model’s performance, considering false positives and negatives. A higher F1 score indicates better overall performance.

(7) $$F1=2\times \frac{P\times R}{P+R}$$

Confusion Matrix: It summarizes the model’s performance by showing the number of true positives, true negatives, false positives, and false negatives. The confusion matrix provides a detailed breakdown of the model’s predictions and errors.

Receiver Operating Characteristic (ROC) Curve and Area Under the Curve (AUC): The ROC curve plots the true positive rate against the false positive rate at various threshold settings. AUC represents the area under the ROC curve and measures the model’s ability to distinguish between pest and non-pest images.

### 3.6. Deployment and Monitoring

Once a satisfactory model is obtained, it can be deployed in production environments for real-time tomato pest classification. The system may also incorporate monitoring mechanisms to track model performance over time and retrain the model periodically with new data to ensure continued accuracy and effectiveness. Deployment of the tomato pest image classification system involves implementing the trained model into production environments, such as on-premises servers, cloud platforms, or edge devices. This typically involves setting up APIs or user interfaces to interact with the model, ensuring compatibility with input data formats, and handling preprocessing steps. Effective deployment ensures that the model can be seamlessly integrated into existing agricultural workflows, providing timely insights for pest management. Monitoring ensures the ongoing performance and reliability of the deployed tomato pest image classification system. This involves tracking key metrics such as inference latency, accuracy, and resource utilization. Monitoring tools and logging mechanisms are essential for detecting and addressing anomalies or failures in real time, ensuring that the classification system operates optimally. Regular monitoring enables proactive maintenance and allows iterative model or deployment configuration improvements based on user feedback and changing environmental conditions.

## 4. Experiment Results and Analysis

**Figure 4:**
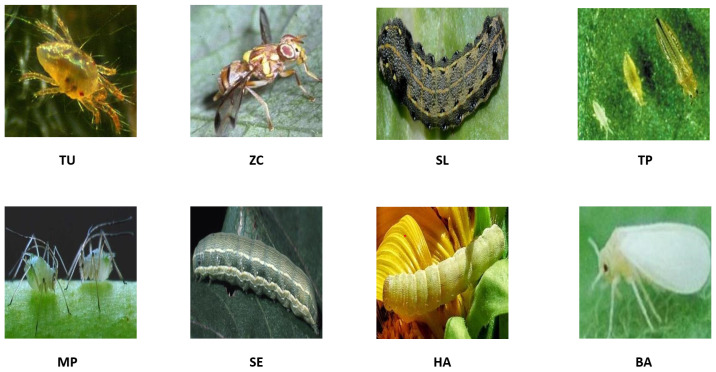
Sample images of tomato pest dataset.

**Figure 5:**
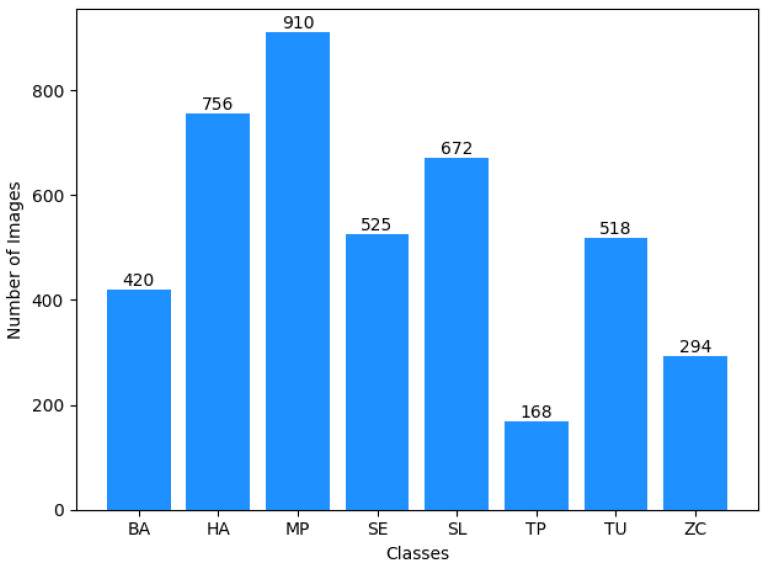
Class-wise sample distribution of tomato pest dataset.

**Figure 6:**
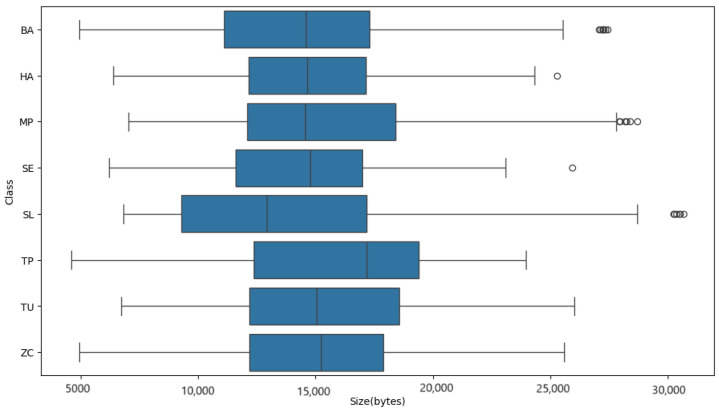
Class-wise image size (in bytes) distribution.

**Figure 7:**
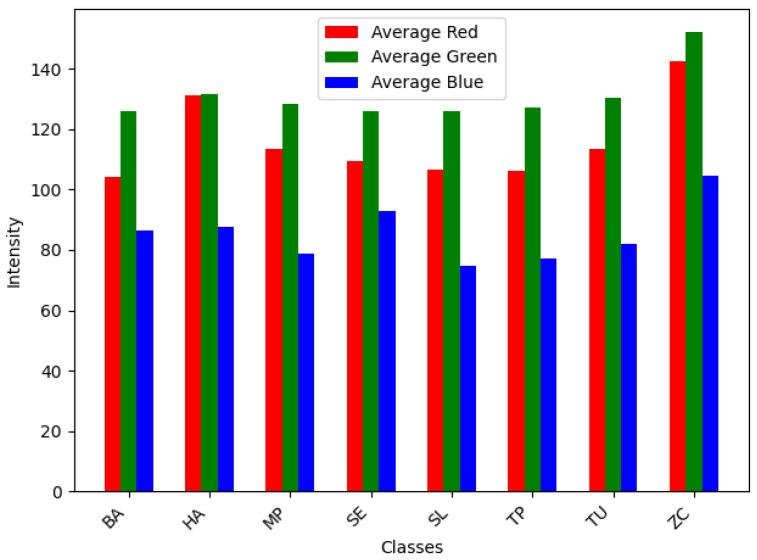
RGB intensity distribution of tomato pest dataset.

A series of experiments are conducted to investigate the classification of tomato pests using images and the impact of different optimizers on model performance. Our study uses a database of eight common tomato crop pests [72], as shown in Figure 4. A total of 4263 images are collected from the database, and the sample class-wise distribution is shown in Figure 5. The tomato pest dataset is imbalanced, and we do not consider balancing the dataset using oversampling in our work. Class TP has the most minor images, around 168, and class MP has the highest number, around 910. The size of images in bytes is plotted classwise in Figure 6, which shows that most classes have images of around 15,000 bytes except for class SL, which has an image size of around 13,000. The RGB intensity distribution shown in Figure 7 implies that every class has high green intensity images as the background is tomato leaves. However, this is irrelevant to our work as we convert the images to grayscale. The images are 299 × 299 × 3 dimensions, converted to 297 × 297 × 1 (grayscale) and passed into the training and testing pipeline that standardizes the image pixel values to avoid exploding gradient problems and easy computation. The machine learning models are trained on Google Colab processor Intel(R) Xenon (R) images with 2 CPU cores, 2.30 GHz CPU frequency, and 12 GB RAM. Sklearn version 1.2.2 [77] is used for all model training and testing of the tomato pest dataset. The following subsection discusses our experiment results based on machine learning models and the impact of different optimizers in a CNN model for classifying tomato pests and comparison with state-of-the-art deep learning models.

### 4.1. Tomato Pest Classification Using Machine Learning Models

Several machine learning models can be utilized for tomato pest classification, each with its advantages and suitability depending on the specific task requirements. Our study analyzed multiple machine learning models for tomato pest classification, including logistic regression, support vector machine (SVM), decision tree, random forest, naive Bayes, and K-nearest neighbors (kNN). The choice of a machine learning model depends on factors such as the task complexity, data availability, computational resources, and the desired level of interpretability and accuracy. Experimentation and evaluation are essential to determine the most suitable model for an undertaken task. Table 2 shows the performance comparison of different machine learning models for tomato pest classification.

**Figure 8:**
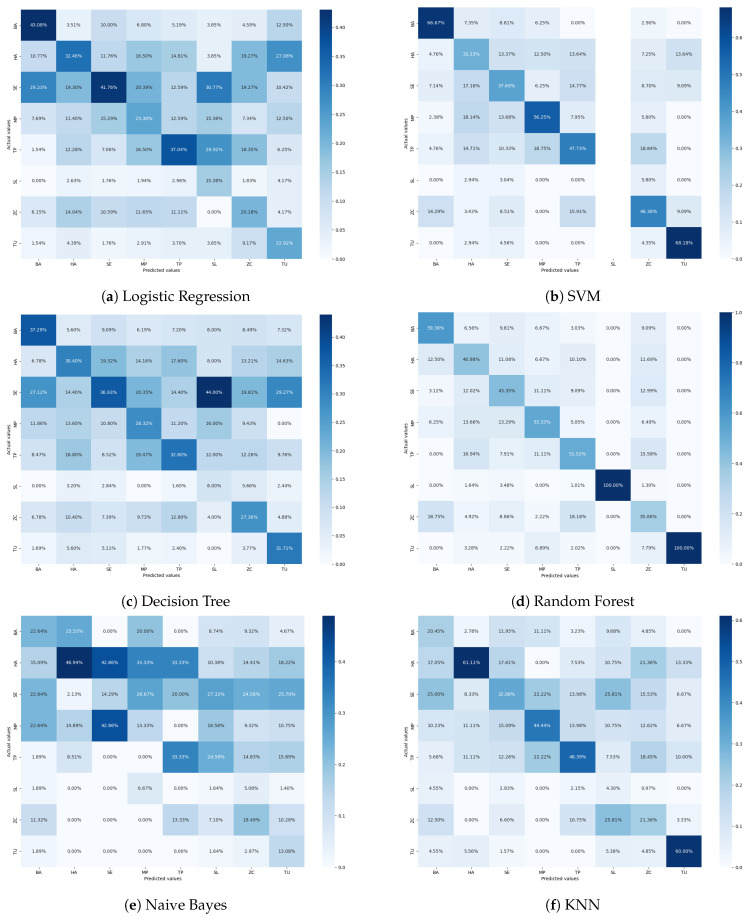
Confusion matrices of different machine learning models for tomato pest classification.

Logistic regression is commonly used for binary classification. However, we implemented logistic regression using a one-versus-rest class strategy with a cross-entropy as a loss function with maximum iterations of 1000 and penalty as none [77]. The results show that the logistic regression has reached an accuracy of 32% with precision, recall, and F1 scores of around 30% (Table 2). The AUC score for logistic regression reached 0.66. For the SVM classifier, we implemented Sklearn’s SVM with ‘rbf’ as the kernel. The SVM fits a plane through data points to classify all classes, which is considered a better non-linear model for classification. The accuracy of SVM on the tomato pest dataset is 41%, and precision, recall, and F1 scores ranged between 45 and 35%. The average AUC score of SVM is 0.81. SVMs are not accurate for imbalanced datasets. Cross-validation techniques or over-sampling can be performed to reduce this impartial classification. Decision tree classifiers follow simple rules inferred from data points that are unsuitable for image data, as the image data are complex and difficult to generalize for models. We have set the depth of the tree as default, i.e., all the possible depths of the decision tree to fit the image data are explored. The decision tree classifier has resulted in an accuracy of 31%, with precision, recall, and F1 score of around 29% (Table 2). Random forest is an ensemble classifier consisting of multiple decision trees. It uses averaging over prediction to reduce overfitting. This classifier achieved the highest accuracy of 46%, precision of 60%, recall of 38%, F1 score of 42%, and the average AUC score of 0.81. Random forest is the best-fitted model under the mentioned experimental conditions, but it still needs thorough hyperparameter tuning and analysis to be finalized. The model can easily handle complex relationships and non-linearity. The naive Bayes machine learning model is well known for text classification tasks and uses the probability of classes instead of features to predict each class image based on the Bayes theorem. The naive Bayes classifier assumes that the images of each class are independent, which is highly violated in real-life scenarios; this makes the classifier perform poorly on image classification. Therefore, a significantly poor performance was observed with a maximum accuracy of 14%. The k-nearest neighbors classifier uses the ‘k’ nearest neighbors of each data point to classify or cluster those nearest points together into a class. We used the KNN classifier from [77] with k = 5. It showed an accuracy of 31%, precision of 28%, 27% recall, F1 score of 27%, and an AUC score of 0.68. Furthermore, we analyzed the performance of each machine learning model based on the confusion matrix, which is shown in Figure 8.

The confusion matrix results from logistic regression show that the BA class has the least misclassification at 43.08% and class SE at 41.78%. Class SL is the most misclassified class, with only 15.38%. Class SL is misclassified as 26.92% TP, 30.77% SE, and 15.38% as MP. The confusion matrix result conveys that classes BA and TU are correctly classified with 66.67% and 68.18%. The class SL has not even been classified as it contains minimal samples. SVM machine learning model results show that the model failed to perform well for the tomato pest classification. The class imbalance in the tomato pest dataset results in a high level of classification error when the system uses the SVM model. The confusion matrix results from the decision tree classification model indicate that class BA and SE are correctly classified as 37.29% and 36.93%. Class SL has the most misclassification of 44% as class SE and 16% as class MP. The confusion matrix results from the random forest machine learning model show that class SL and TU are 100% correctly classified. The most misclassified class was ZC, which was 15.58% and 12.99% classified as BA and TP classes. The confusion matrix of the naive Bayes classifier shows high variation in the classification of each class. Class HA is correctly classified as 48.94%, and class MP has 42.86% accuracy. The most misclassified classes are SL at 27.32% as SE and 24.59% as TP. Class MP is 33.33% misclassified as HA and 26.67% as class SE. The KNN model classified class HA as 61.11% and TU class as 60% correctly. The confusion matrix results of KNN show that class SL is highly misclassified as ZC and SE at 25.81% each. The confusion matrix results from machine learning models reveal that classes like SL and HA show the highest misclassification errors. Visual similarities between these pests, such as shared features like body shape, texture, coloration, and overlapping environmental contexts, make differentiation challenging. Dataset imbalance, where certain classes are over- or underrepresented, can skew the model’s learning and hinder its ability to generalize distinct features. Additionally, low-quality images, including blurry or occluded visuals, and variations across pest life stages, such as larvae, pupae, and adult forms, can further complicate classification. Limitations in the model’s feature extraction, such as insufficient granularity or inaccurate focus on critical regions, also contribute to errors. To address these issues, strategies like data augmentation, enhanced feature localization through methods like Grad-CAM, improved labeling and preprocessing, and expanding the dataset with diverse samples of these pests can significantly improve model performance. Incorporating attention mechanisms or hybrid models can refine class distinction, reduce misclassifications, and enhance classification accuracy.

**Figure 9:**
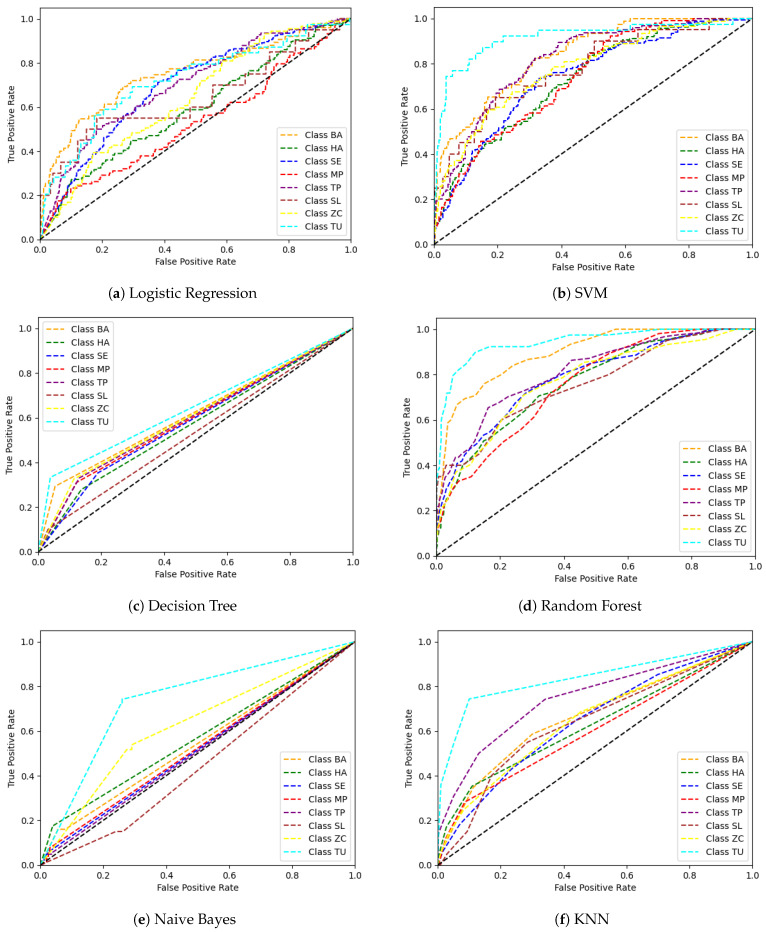
ROC curves of different machine learning models for tomato pest classification.

Next, we investigated the classification performance of machine learning models based on the ROC curve, which is useful information when the dataset has a class imbalance. Figure 9 shows the ROC curves of multiple machine learning models for tomato pest classification. The ROC curve for the logistic regression model shows that the BA class true positives are correctly classified compared to other classes, while class MP has the lowest classification rate. The SVM ROC curve shows that class TU has a high true positive and false negative rate. Class MP has the lowest classification rate, but the rate of classification by SVM shows better performance than other models. In contrast, the confusion matrix results show that it has struggled due to the imbalanced sample distribution of the dataset. The ROC curve of the decision tree model indicates that class TU has a high correct classification rate and class SL has the lowest classification rate. The ROC curve describes the decision tree classifier has a poor discrimination ability to classify positive and negative instances due to the class imbalance from the tomato pest dataset. The ROC curve for random forest demonstrates that class TU is more correctly classified, while class SL has the lowest classification rate, along with class MP. The ROC behavior of random forest hints at a non-linear relationship between the instances and possible model overfitting. The ROC curve for the naive Bayes classifier shows that the classification rate is significantly less, and class SL is under the baseline of random guess classification. This behavior is highly undesirable as the model struggles to differentiate between positive and negative instances from a random guess scenario. The ROC curve of the KNN classifier validates that class TU has a high positive/negative classification rate while class MP has the lowest classification rate. The ROC curve behavior suggests that the model struggles to differentiate between positive and negative instances of each class.

### 4.2. Impact of Various Optimizers on the Classification of Tomato Pest Images Using CNN Model

To evaluate the impact of various optimizers on the classification of tomato pest images using a CNN model, we conducted a series of experiments with different optimizers on a curated dataset. We began by hyperparameter tuning the CNN model for each optimizer. Hyperparameter tuning is crucial for finding the best parameters that result in the highest model performance. Several techniques for parameter tuning include grid search, random search, and Bayesian optimization. We used Bayesian optimization to tune each optimizer’s learning rate and L2 regularizer. In Bayesian optimization, random parameters are initially selected, and subsequent iterations focus on exploring the best parameters in the search space until the global maximum of validation accuracy is reached. Bayesian optimization uses active learning to avoid the most uncertain search spaces and explore the best regions of the objective function. We used a learning rate search space between $10^{-2}$ and $10^{-4}$. The L2 regularizer search space was defined between $10^{-3}$ and $10^{-5}$. We set the maximum number of trials to five and trained the model for 100 epochs with a batch size of 64. Table 3 summarizes the hyperparameter tuning experiments for the CNN model with various optimizers.

In Table 3, the Adam optimizer achieved the highest validation accuracy of 83.89% with a learning rate of 0.001 and an L2 regularizer value of 0.0001. Attempts to improve Adam’s performance with lower learning rates and regularizer values did not yield better results. The AdaDelta optimizer, using a learning rate of 0.0001 and an L2 regularizer value of 0.001, resulted in a significantly lower validation accuracy of 24.28%. As shown in Table 3, AdaDelta did not perform well on the tomato pest dataset with other hyperparameter combinations. The AdaGrad optimizer, with a learning rate of 0.0001 and an L2 regularizer of 0.0001, achieved a validation accuracy of 24.67%. The RMSprop optimizer achieved the best performance, with a validation accuracy of 85.84%, using a learning rate of 0.001 and an L2 regularizer of 0.0001. However, when trained with a learning rate of 0.0001 and an L2 regularizer of 0.001, RMSprop had the lowest validation accuracy of 17.76%. The SGD optimizer trained with a learning rate of 0.001 and an L2 regularizer of 0.0001 achieved a validation accuracy of 24.41%. Other hyperparameter settings for SGD did not improve its performance, with a global minimum validation accuracy of 23.89%. The Nadam optimizer, with a learning rate of 0.001 and an L2 regularizer of 0.0001, achieved a validation accuracy of 85.45%. From the hyperparameter tuning results summarized in Table 3, RMSprop stands out with the highest validation accuracy of 85.84%, followed by Nadam at 85.45% and Adam at 83.89%. These three optimizers share an adaptive learning rate strategy, which allows them to adjust the learning rate dynamically and converge towards maximum accuracy, thereby achieving superior performance compared to other optimizers. The adaptive learning rate feature in the Adam, Nadam, and RMSprop optimizers is crucial in their exceptional performance. This capability enables the optimizers to adjust the learning rate based on the gradients’ magnitudes, leading to more efficient and stable convergence. The results indicate that this adaptive mechanism is particularly effective for the tomato pest classification. In contrast, optimizers like AdaDelta and AdaGrad, which did not perform well, lack the same degree of adaptability in their learning rates or suffer from issues like overly aggressive updates that can lead to poor convergence. The significant drop in validation accuracy for these optimizers highlights the importance of selecting an optimizer that can handle the nuances of the dataset and the model’s architecture. SGD, despite being a widely used optimizer, showed relatively poor performance in tomato pest classification. This outcome underscores the challenges of using a fixed learning rate, which can lead to slower convergence and difficulty in escaping local minima. These experiments highlight the importance of hyperparameter tuning and choosing the right optimizer for tomato pest classification. The adaptive learning rate strategies of RMSprop, Nadam, and Adam have proven to be particularly effective for classifying tomato pest images, offering a robust approach to achieving high validation accuracy.

**Figure 10:**
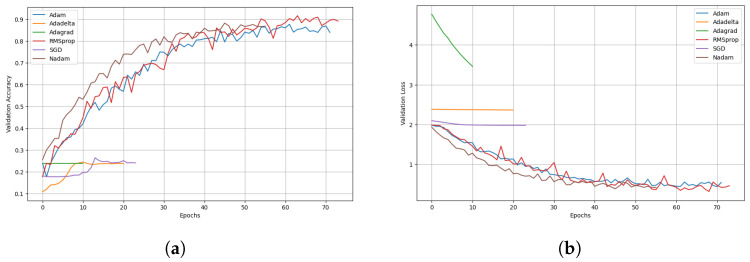
Validation accuracy and loss curves of the CNN model trained with different optimizers. (**a**) Validation accuracy curves of each optimizer. (**b**) Validation loss curves of each optimizer.

Furthermore, we conducted an in-depth analysis of the performance of each CNN optimizer based on validation accuracy and loss. Figure 10 illustrates the CNN model’s validation accuracy and loss curves trained with optimizers over 100 epochs, with an early stopping criterion based on constant validation accuracy. In Figure 10a, we observe the trends in validation accuracy. The validation accuracy for the RMSprop, Nadam, and Adam optimizers steadily increases and converges around 70 epochs. This indicates a stable learning process and effective convergence towards optimal performance. The AdaGrad optimizer maintains a constant validation accuracy of approximately 20% for the initial ten epochs. The training is terminated early due to the early stopping criterion triggered by the lack of improvement in validation accuracy. In AdaDelta, the validation accuracy increases to ten epochs and then plateaus, indicating early convergence. The SGD optimizer initially shows a constant validation accuracy but starts improving after around 12 epochs, terminating at 22 due to the early stopping criterion based on constant validation accuracy. The convergence behavior across these optimizers enhances understanding of the CNN model’s performance. The optimizers’ unique gradient update mechanisms influence the differences in convergence speeds; Nadam and AdaDelta converge faster than RMSprop. Nadam, combining the Nesterov-accelerated gradient with Adam’s adaptive learning rate adjustments, benefits from both momentum and smoother updates, allowing faster convergence in fewer epochs. AdaDelta, with its adaptive learning rate per parameter, similarly enables quicker adjustments during training, which helps reach a stable solution faster, albeit sometimes with less precision in final accuracy. In contrast, RMSprop, while effective in maintaining stable learning by adapting the learning rate for each parameter, typically converges more slowly in our model due to its reliance on the average of squared gradients alone, which can occasionally lead to smaller steps during each iteration.

Figure 10b presents the validation loss comparison for each optimizer. The validation loss for the RMSprop, Nadam, and Adam optimizers decreases and reaches a convergence point of around 70 epochs, aligning with their validation accuracy trends. The AdaDelta and SGD optimizers exhibit a constant validation loss for up to twenty epochs. AdaDelta shows early convergence, whereas SGD shows improvement after an initial plateau. Despite decreasing validation loss, AdaGrad’s validation accuracy remains constant. Training is terminated after ten epochs due to the early stopping criterion based on validation accuracy rather than loss. These observations highlight the varying effectiveness of different optimizers in training CNN models for tomato pest classification. Table 4 summarizes the classification performance of various optimizers used in training CNN models for tomato pest classification.

The results in the Table 4 demonstrate a clear performance variation among optimizers used in training CNN models for tomato pest classification. RMSprop and Nadam emerge as the most effective optimizers, achieving the highest training and validation accuracies of 91.72% and 89.09% for RMSprop and 91.62% and 86.75% for Nadam, respectively. Both optimizers also exhibit competitive precision, recall, and F1 scores. In contrast, optimizers like AdaDelta, AdaGrad, and SGD show significantly lower performance across all metrics, with validation accuracies below 25% and F1 scores in the 5 to 6 range, indicating poor generalization and convergence. Adam demonstrates decent performance with a validation accuracy of 83.89% and an F1 score of 84%, positioning it as a viable alternative to RMSprop and Nadam. In our analysis, RMSprop emerged as the most effective optimizer for the proposed CNN model, achieving the highest validation accuracy. Nadam and Adam also performed well, with validation accuracies close to RMSprop. The common feature among these top-performing optimizers is their adaptive learning rate mechanism, which helps avoid poor parameter search spaces and ensures smoother optimization. This adaptability proves crucial for achieving high performance in tomato pest classification tasks, highlighting the importance of selecting the appropriate optimizer for training CNN models.

**Figure 11:**
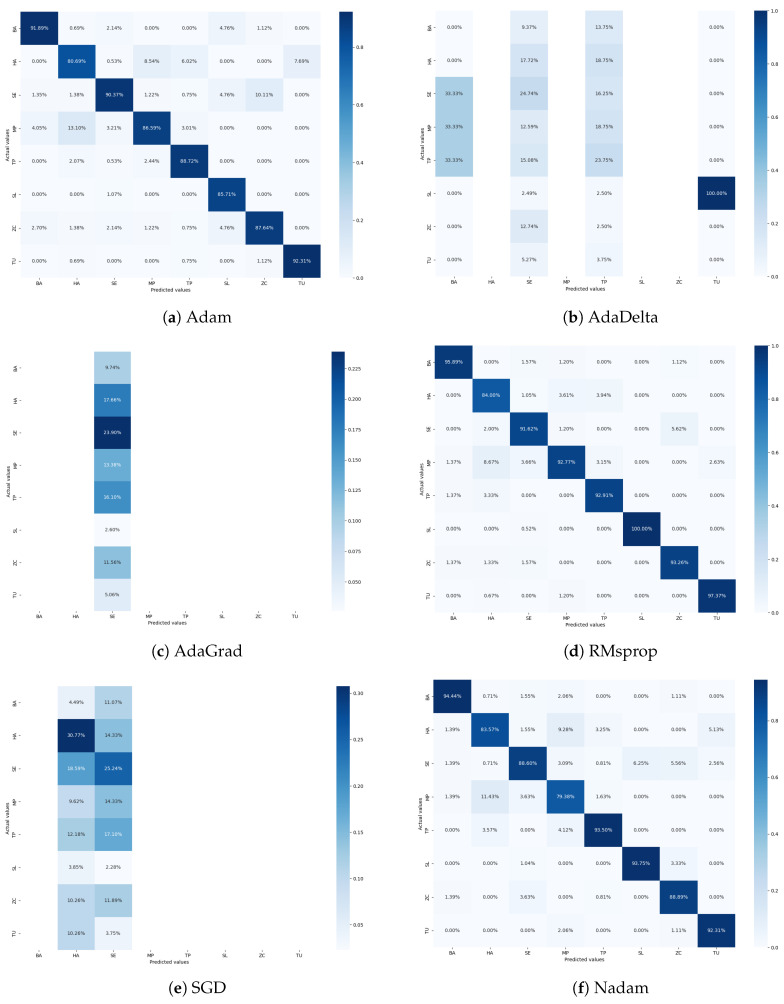
The confusion matrix results of the CNN model with different tuned optimizers.

For further analysis, Figure 11 presents the confusion matrices illustrating the performance of the CNN model with various tuned optimizers. The confusion matrix for the Adam optimizer showed the least accuracy for class HA, with 80.69% correctly classified samples. It also indicates misclassifications of 8.54% as class MP and 6.02% as class TP. This suggests confusion between these classes, potentially due to similarities in their visual features. The confusion matrix for the AdaDelta optimizer demonstrates a significant imbalance in classification, reflecting its poor performance (Table 4). The misclassifications and low accuracy rates indicate that AdaDelta struggles to learn the distinguishing features of tomato pest classes. As depicted in Figure 11c, the AdaGrad optimizer’s confusion matrix shows that only class TU is correctly classified, while all other classes remain undetected. This starkly highlights AdaGrad’s inadequate performance on the tomato pest dataset, which cannot differentiate between the classes effectively. The RMSprop optimizer’s confusion matrix performs better, with class HA having the least accuracy at 84%. There are minor misclassifications, with approximately 3% of samples misclassified as class MP and TU, respectively. Class SL has a perfect classification rate of 100%, underscoring RMSprop’s robustness in certain categories. The confusion matrix for SGD reveals poor performance, with only classes HA and SE being correctly classified. The substantial misclassification and low accuracy reflect SGD’s inefficacy in training the proposed CNN model on the tomato pest dataset. The Nadam optimizer’s confusion matrix shows that class MP is the most misclassified, with 11.43% of its samples classified as class HA. Class HA is also significantly misclassified as class MP at a rate of 9.28%. Nadam and RMSprop exhibit the most minor misclassified samples despite these misclassifications.

The confusion matrices indicate a consistent pattern across all optimizers, where class HA is frequently misclassified as class MP. This suggests a high visual similarity between these two classes, complicating accurate classification. RMSprop and Nadam stand out for their higher overall accuracy and fewer misclassifications, demonstrating their effectiveness. Figure 11c presents the training time taken by each optimizer, measured in seconds. The results indicate significant variations in the convergence times of the different optimizers.

**Figure 12:**
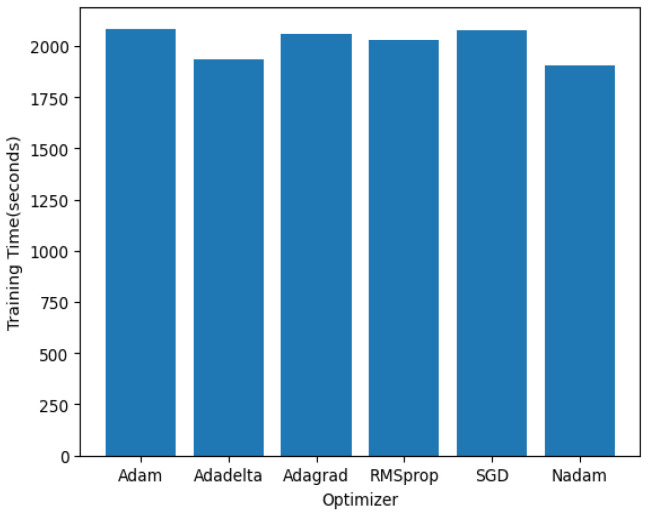
Training time taken by each optimizer.

The training time analysis depicted in Figure 12 underscores the trade-offs between convergence speed and computational complexity for different optimizers. The AdaDelta, AdaGrad, RMSprop, and Nadam optimizers demonstrate similar training times. The comparable training durations can be attributed to their adaptive learning rate mechanisms, which require additional computational resources per iteration. This similarity suggests that these optimizers’ underlying complexities and computational overheads are alike, leading to an almost parallel performance in training time. The Nadam optimizer, however, stands out with a significantly lower training time compared to AdaDelta, AdaGrad, RMSprop, and Adam. Nadam’s efficient combination of the advantages of the Adam and RMSprop optimizers and its adaptive learning rate adjustments allow it to converge faster. This efficiency makes Nadam preferred in scenarios where accuracy and training speed are crucial. On the other hand, AdaDelta converges the fastest, requiring only 1933 s to complete the training. The quicker convergence of AdaDelta is primarily due to its decaying average of past gradients, which involves less computation per iteration than the adaptive learning rate methods. This simplicity allows AdaDelta to process each batch of data swiftly, making it highly efficient in terms of time.

**Figure 13:**
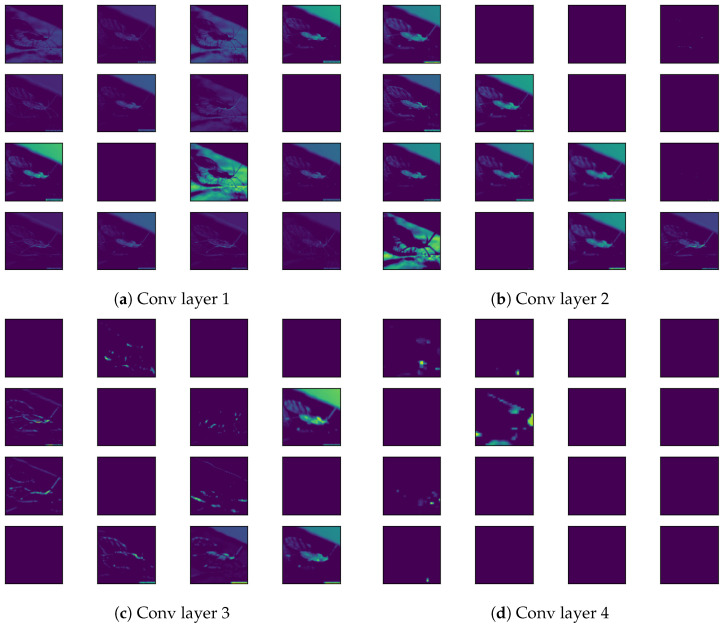
Feature map visualization of our proposed CNN.

In the proposed CNN for tomato pest classification, we comprehensively analyzed the feature map visualizations to better understand the impact of CNN layers on classification accuracy. Feature maps represent higher-level input image abstractions generated through consecutive convolution operations. These visualizations offer valuable insights into the internal workings of the CNN by highlighting the key regions of activation that influence the model’s decision-making process. Feature maps essentially reveal the important features, such as edges, textures, and specific regions, that the CNN layers capture as the image data pass through the network. By visualizing these maps, we gain a clearer understanding of the patterns learned by the network and how each layer progressively refines the image representation to contribute to accurate classification. The feature map visualizations for the proposed CNN, which consists of four convolutional layers, are depicted in Figure 13. The first convolutional layer generates 32 feature maps, each with a resolution of 297 × 297 pixels. These feature maps capture the initial, basic features of the image, such as simple edges and textures. When overlapped and reduced to 16 images, the feature maps reveal that the CNN has identified elementary details, such as the outline of the pest and some background information, including the leaves on which the pest is situated. This layer acts as the foundational stage in the feature extraction, detecting the most rudimentary patterns in the input image. Moving deeper into the network, the second convolutional layer generates 64 feature maps, each with a resolution of 146 × 146 pixels. After overlapping and reducing these maps to 16, we observe that this layer is more focused on detecting specific features of the pest, including its body parts, rather than merely its edges. This layer begins to form a more holistic representation of the pest, moving beyond simple shapes and lines to capture more informative features of the image. The third convolutional layer further increases the complexity of the extracted features, generating 128 feature maps with a resolution of 71 × 71 pixels. After reduction to 16 feature maps, this layer highlights more intricate patterns, such as combinations of edges and textures that define the pest’s structure. This convolutional layer is crucial in detecting medium-level features, which are more abstract than those identified by the previous layers. It starts to distinguish different regions of the pest’s body and the interplay between various patterns in the image. Finally, the fourth convolutional layer produces the highest number of feature maps, generating 256 maps with a resolution of 33 × 33 pixels. These feature maps are resized to 16 and reveal the image’s most complex and high-level features. This layer captures the most relevant and essential pest image regions critical for accurate classification. The final feature maps produced by this layer intensely focus on the pest’s key areas that the model uses to make its classification decision, filtering out irrelevant background information and noise. The visualization of these feature maps highlights how the proposed CNN model effectively transforms the raw input image through a series of convolutional operations, gradually refining the features until the most salient aspects of the pest image are isolated. Each layer contributes to the extraction of increasingly complex patterns, starting with basic shapes and progressing to detailed textures and high-level features crucial for precise classification. This process underscores the power of deep learning in handling complex image data, enabling the model to automatically learn and prioritize the most critical features for accurate pest identification. Through this analysis, we gain a deeper understanding of how the CNN leverages hierarchical feature extraction to successfully classify tomato pest images, aiding in developing more efficient and reliable automated pest detection systems for agricultural applications.

We further analyzed the performance of various optimizers using CNNs with different cross-validation techniques to ensure model generalization. Cross-validation is crucial for assessing how well the proposed learning models can perform on unseen data. Specifically, we employed k-fold cross-validation, where the dataset is divided into k number of folds, and the model is trained and validated on each fold separately. This method provides a comprehensive evaluation by ensuring that each data point is used for training and validation. We applied five-fold cross-validation to the tomato pest dataset using the proposed CNN model with different optimizers. This approach ensures that the model’s performance is not overly dependent on a specific subset of the data, thereby improving its generalization capabilities in real-world scenarios. Table 5 presents each optimizer’s mean validation accuracy and F1 score across the five cross-validation folds. With a learning rate of 0.001, the Adam optimizer achieved a mean validation accuracy of 48.86% and an F1 score of 42.96%, indicating moderate performance across the folds. The AdaDelta optimizer, with its optimal learning rate of 0.0001, yielded a mean validation accuracy of 18.05% and a significantly lower F1 score of 8.33%, highlighting its poor performance on this dataset. The AdaGrad optimizer, with a learning rate of 0.001, resulted in a mean validation accuracy of 21.53% and the lowest F1 score of 7.64% among the optimizers tested, suggesting limited effectiveness in handling the tomato pest classification task. The RMSprop optimizer, with a learning rate of 0.001, demonstrated better performance with a mean cross-validation accuracy of 64.64% and an F1 score of 58.81%, positioning it as a strong contender for this task. The SGD optimizer, with a learning rate of 0.001, achieved a mean validation accuracy of 21.4% and an F1 score of 8.07%, further underscoring its limited effectiveness in this scenario. Notably, the Nadam optimizer, with a learning rate of 0.001, delivered the best performance with a mean validation accuracy of 79.12% and an F1 score of 78.92%. The cross-validation results highlight Nadam as the most effective optimizer for the proposed CNN model in tomato pest classification, especially considering the F1 score as a key performance metric. This finding suggests that Nadam offers a robust and reliable solution for this challenging agricultural image classification task.

**Figure 14:**
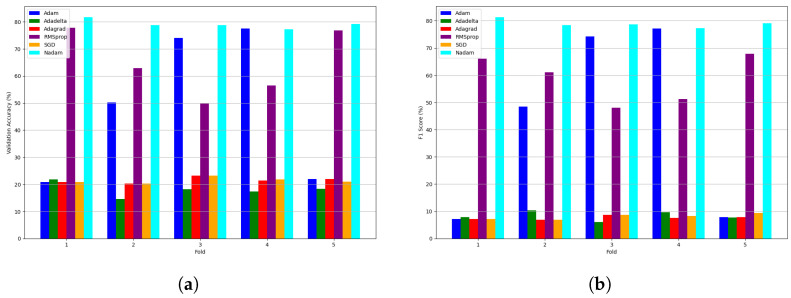
Cross-validation results of optimizers compared for each fold. (**a**) Cross-validation accuracy comparison of each optimizer. (**b**) Cross-validation F1 score comparison of each optimizer.

Figure 14 provides a comparative analysis of the validation accuracy and F1 scores achieved by different optimizers during the cross-validation of the proposed CNN model on the tomato pest dataset. The results illustrate the performance across five distinct folds, offering insights into each optimizer’s consistency and effectiveness. In terms of validation accuracy from Figure 14a, Nadam consistently outperforms the other optimizers across all five folds, demonstrating remarkable stability and reliability. This consistent performance highlights Nadam’s robustness in handling the variability within the dataset, making it the most reliable optimizer for this classification task. Conversely, RMSprop, which achieves the second-highest validation accuracy overall, exhibits a noticeable decline in performance during the third and fourth folds. This dip indicates potential sensitivity to certain portions of the dataset, suggesting that while RMSprop is generally effective, it may struggle with specific data distributions or outliers. The Adam optimizer, while securing the third-highest validation accuracy, shows significant variability in its performance, particularly during the first and fifth folds. This inconsistency raises concerns about Adam’s generalization capabilities, as its performance appears highly dependent on the specific data used for training and validation in each fold. The fluctuating results suggest that Adam may require further fine-tuning or may not be as robust as Nadam and RMSprop in this particular application. In Figure 14b, the F1 scores further corroborate the validation accuracy findings. Nadam again emerges as the leading optimizer with consistent and high F1 scores across all folds, reinforcing its effectiveness in balancing precision and recall. RMSprop maintains the second-highest F1 score despite its variability in validation accuracy. This suggests that RMSprop can still maintain a reasonable balance between precision and recall, even when its overall accuracy fluctuates. However, Adam fails to perform well in terms of the F1 score, indicating that its predictions are less reliable and balanced compared to Nadam and RMSprop. The other optimizers—AdaDelta, AdaGrad, and SGD—exhibit F1 scores below 10%, reflecting their limited capacity to classify tomato pests in this dataset effectively. Their poor performance underscores these optimizers’ challenges when applied to complex and imbalanced datasets such as the one used in this study. The results from Figure 14 give clear evidence of the superior performance of Nadam in both validation accuracy and F1 score, highlighting its consistent and reliable performance across all folds. RMSprop, while effective, shows some vulnerability to specific folds, and Adam’s inconsistency further diminishes its suitability for this task. These findings emphasize the importance of selecting the right optimizer for CNN models in agricultural image classification. Nadam is this study’s most robust and reliable choice for tomato pest classification.

### 4.3. Comparison with State-of-the-Art Deep Learning Models

The proposed CNN-optimized approaches were benchmarked against several state-of-the-art deep learning models, including LeNet, AlexNet, Xception, Inception, ResNet, and MobileNet, to evaluate their effectiveness in classifying tomato pests. The performance comparison of different deep learning models with the proposed CNN-optimized approaches, as presented in Table 6, highlights the significant advancements achieved by the proposed methods.

Among the state-of-the-art models evaluated, Inception and ResNet exhibited the highest performance, achieving an accuracy of 69%. Inception also demonstrated strong precision (76%) and F1 score (62%), while ResNet achieved an F1 score of 67%. MobileNet followed closely with an accuracy of 66%, showcasing balanced precision, recall, and F1 scores (66%, 65%, and 63%, respectively). However, the proposed CNN optimized with the RMSprop optimizer outperformed all these models, achieving the highest accuracy of 89.09% and an F1 score of 86%. This improvement can be attributed to an optimized training process, which leveraged RMSprop’s ability to handle variable learning rates effectively. Furthermore, the proposed CNN optimized with the Nadam optimizer, evaluated using cross-validation, achieved a mean accuracy of 79.12% and an F1 score of 78.92%, demonstrating its robustness and consistency across folds. In contrast, other models like LeNet, AlexNet, and Xception underperformed relative to the proposed approaches. LeNet achieved an accuracy of only 39.5%, while AlexNet, despite being a foundational deep learning architecture, exhibited the poorest performance with an accuracy of 24% and an F1 score of just 5%. Xception, with its advanced depth-wise separable convolutions, achieved moderate results but could not surpass the effectiveness of the proposed approaches. These results emphasize the importance of optimizer selection and model customization in achieving superior performance for specific tasks such as tomato pest classification. The RMSprop-optimized CNN particularly demonstrates its capability to manage challenges like class imbalance and variability in pest images, setting a new benchmark for accuracy and reliability in this domain.

**Figure 15:**
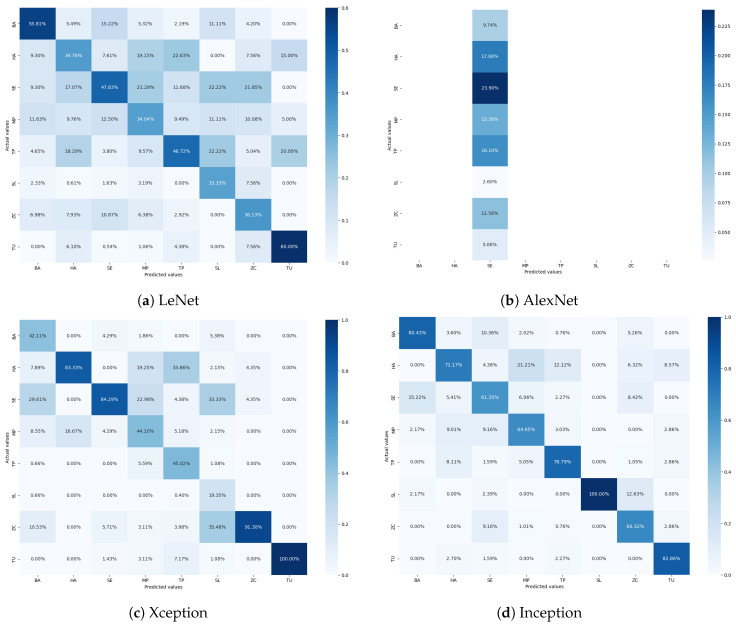

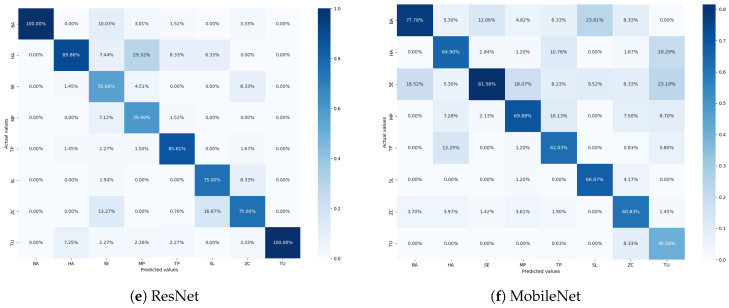
Confusion matrices of different deep learning models for tomato pest classification.

The confusion matrix of the deep learning models, as depicted in Figure 15, provides a detailed breakdown of classification performance for each pest class. The analysis reveals that the AlexNet model failed to differentiate between the tomato pest classes, with all instances being misclassified as the SE class, highlighting its inefficacy for this task. Similarly, the LeNet model exhibited significant misclassification errors, particularly for the SL and HA classes, indicating challenges in distinguishing these pests. The Xception model showed improved classification for the HA class compared to LeNet but struggled with accurate identification of the SL class. Additionally, the BA class had the poorest performance under the Xception model, indicating specific weaknesses in handling certain pest categories. While performing better overall, the Inception model had SE as its lowest-performing class, reflecting a consistent difficulty among models in accurately identifying this pest type. ResNet demonstrated robust performance for most pest classes but struggled with poor classification accuracy for the SE and MP classes. MobileNet, known for its lightweight architecture, showed its lowest classification performance for the TU and ZC classes, suggesting room for improvement in capturing features unique to these pests. Overall, these results underscore the varying strengths and limitations of each deep learning model in tomato pest classification. The consistent misclassification patterns in certain pest classes suggest the need for targeted optimization strategies or class-specific augmentation to enhance model performance.

## 5. Challenges and Limitations of the Tomato Pests Classification System

Tomato pest classification systems encounter several challenges that hinder their effectiveness in real-world agricultural applications. These challenges include pest variability, image quality issues, environmental conditions, limited annotated datasets, generalization difficulties, and resource constraints. To address the variability in pest morphology and overlapping features, data augmentation techniques such as flipping, cropping, and rotation are applied to simulate variations and improve model robustness. Poor image quality caused by field conditions, such as low lighting, occlusions, or cluttered backgrounds, is mitigated through preprocessing methods like contrast enhancement, noise reduction, and normalization. Variability in environmental conditions, such as lighting and weather changes, which impact pest appearance and classification accuracy, is tackled using domain adaptation techniques and fine-tuning models with region-specific data. Additionally, incorporating metadata such as time of capture and geographical location enhances model contextual understanding.

Limited annotated datasets are addressed through data augmentation, synthetic data generation, and transfer learning using pre-trained CNNs, which allow models to leverage knowledge from large, generic datasets. Overfitting, a common challenge when working with small datasets, is minimized through strategies such as dropout, early stopping, and regularization techniques, alongside careful cross-validation to ensure generalization. Furthermore, transfer learning combined with region-specific re-training enhances model adaptability to new environments with different pest species and agricultural practices. To ensure detection sensitivity for underrepresented pest species or stages, targeted re-training with class-weighted loss functions is employed, improving recognition for subtle or less prevalent cases.

Efficient and lightweight architectures like MobileNet are utilized to create mobile-compatible models, ensuring scalability and accessibility for farmers and agricultural stakeholders with limited resources. Integration into existing farming workflows is facilitated by developing user-friendly interfaces and offering recommendations for pest management strategies, such as biological control or pesticide application. Resource constraints are mitigated by leveraging cloud-based computing platforms, enabling high-performance processing without requiring local infrastructure.

Ethical and regulatory considerations are also central to the development of these systems. Ensuring data privacy, adhering to environmental sustainability guidelines, and complying with local agricultural regulations are vital for responsible deployment. By addressing these challenges through targeted solutions, tomato pest classification systems are made more robust, scalable, and practical, offering a valuable tool for enhancing crop health, productivity, and sustainable pest management in real-world agricultural settings.

## 6. Conclusions

Classifying tomato pests using images presents a significant challenge in agriculture, with implications for crop management and yield optimization. In this study, we explored the application of the CNN model for automating the classification of tomato pest images and investigated the impact of different optimizers on model performance. By leveraging deep learning techniques and carefully selecting appropriate optimizers, we can develop robust and automated pest detection and management solutions, ultimately contributing to increased agricultural productivity and food security. Our experiments demonstrated the efficacy of CNNs in accurately classifying tomato pest images. The CNN model trained on diverse annotated image datasets achieved high classification accuracy, showcasing the potential of deep learning techniques for pest detection and management. We evaluated the performance of different optimizers, including AdaDelta, AdaGrad, Adam, RMSprop, SGD, and Nadam, on tomato pest classification tasks. Our analysis revealed variations in convergence speed, stability, and robustness to hyperparameters among the optimizers. Based on our experiments, the RMSprop optimizer emerged as universally superior for all tomato pest classification tasks when we used the traditional data splitting for training, testing, and validation. The cross-validation results conclude that the Nadam optimizer showed the best results for tomato pest classification. The choice of optimizer depends on factors such as dataset characteristics, model architecture, and computational resources. Practitioners should carefully evaluate and select the optimizer that best suits their requirements. Automated pest detection systems based on CNNs offer promising opportunities for enhancing pest management practices in agriculture. Farmers can implement timely interventions to mitigate crop losses, reduce pesticide usage, and improve overall crop health by accurately identifying tomato pests from images. Continued research and innovation in this field are essential for addressing emerging challenges in crop protection and ensuring the resilience of global food systems.

Future research in automated pest detection can focus on advanced deep learning models like Vision Transformers and hybrid architectures to improve classification performance and scalability. Integrating multi-modal data, such as environmental conditions and crop metrics, with explainable AI techniques can enhance model usability and trust. Developing real-time lightweight models for deployment on edge devices and addressing dataset imbalances with advanced augmentation methods or generative adversarial networks (GANs) will improve system robustness. Expanding datasets, exploring transfer learning for cross-crop applications, and assessing these systems’ economic and environmental impacts can promote their adoption in sustainable agriculture. Integrating these solutions with precision agriculture tools can optimize resource use and minimize crop damage, advancing global food security efforts.

## Figures and Tables

**Table 1:** Summary of CNN model layers and their corresponding output shapes and number of parameters.

Layer Type	Output Shape	Param #
Conv2D	None, 297, 297, 32	320
MaxPooling2D	None, 148, 148, 32	0
Conv2D	None, 146, 146, 64	18,496
MaxPooling2D	None, 73, 73, 64	0
Conv2D	None, 71, 71, 128	73,856
MaxPooling2D	None, 35, 35, 128	0
Conv2D	None, 33, 33, 256	295,168
MaxPooling2D	None, 16, 16, 256	0
Flatten	None, 65,536	0
Dense	None, 512	33,554,944
Dense	None, 256	131,328
Dropout	None, 256	0
Dense	None, 8	2056

**Table 2:** Performance comparison of different machine learning models for tomato pest classification.

Model	Accuracy (%)	Precision (%)	Recall (%)	F1 Score (%)	Average AUC
Logistic Regression	32	30	30	30	0.66
SVM	41	45	34	35	0.81
Decision Tree	31	29	29	29	0.59
Random Forest	46	60	38	42	0.81
Naive Bayes	14	21	22	14	0.56
K-Nearest Neighbors	31	37	28	27	0.68

**Table 3:** Hyperparameter tuning of proposed CNN model with various optimizers.

Optimizer	Trial	L2	Learning Rate	Val. Accuracy
Adam	Trial 1	0.0001	0.01	23.89
	Trial 2	0.01	0.01	23.89
	Trial 3	0.001	0.001	23.89
	Trial 4	0.0001	0.001	83.89
	Trial 5	0.0001	0.0001	68.18
AdaDelta	Trial 1	0.001	0.0001	24.28
	Trial 2	0.01	0.01	24.02
	Trial 3	0.01	0.001	23.76
	Trial 4	0.001	0.001	23.89
	Trial 5	0.01	0.0001	18.7
AdaGrad	Trial 1	0.0001	0.001	24.54
	Trial 2	0.01	0.0001	24.54
	Trial 3	0.0001	0.01	23.89
	Trial 4	0.0001	0.0001	24.67
	Trial 5	0.01	0.01	24.93
RMSprop	Trial 1	0.01	0.01	23.89
	Trial 2	0.001	0.01	23.89
	Trial 3	0.001	0.0001	23.87
	Trial 4	0.0001	0.0001	63.89
	Trial 5	0.0001	0.001	85.84
SGD	Trial 1	0.01	0.0001	17.76
	Trial 2	0.0001	0.001	24.41
	Trial 3	0.01	0.01	23.89
	Trial 4	0.001	0.01	23.86
	Trial 5	0.001	0.001	23.89
Nadam	Trial 1	0.001	0.001	81.03
	Trial 2	0.01	0.0001	56.75
	Trial 3	0.001	0.0001	59.22
	Trial 4	0.0001	0.001	85.45
	Trial 5	0.01	0.001	23.89

**Table 4:** Classification performance of various optimizers used in training CNN models.

Optimizer	Training Accuracy	Training Loss	Validation Accuracy	Validation Loss	Precision (%)	Recall (%)	F1 Score (%)
Adam	89.83	0.3210	83.89	0.5452	83	84	84
AdaDelta	22.98	2.5430	23.77	2.3654	6	12	6
AdaGrad	20.29	3.5144	23.89	3.4608	3	12	5
RMSprop	91.72	0.2963	89.09	0.4637	88	85	86
SGD	20.38	1.9876	24.02	1.9810	3	12	5
Nadam	91.62	0.2812	86.75	0.4192	88	86	87

**Table 5:** Cross-validation accuracy and F1 score for different optimizers.

Optimizer	Fold	Val Acc. (%)	F1 Score (%)
Adam	1	20.78	7.15
	2	50.16	48.42
	3	74.03	74.24
	4	77.44	77.10
	5	21.92	7.88
	**Mean**	48.86	42.96
AdaDelta	1	21.75	7.83
	2	14.61	10.42
	3	18.18	6.10
	4	17.37	9.60
	5	18.34	7.69
	**Mean**	18.05	8.33
AdaGrad	1	20.78	7.15
	2	20.29	6.85
	3	23.21	8.75
	4	21.43	7.56
	5	21.92	7.88
	**Mean**	21.53	7.64
RMSprop	1	77.76	66.00
	2	62.81	61.04
	3	50.00	48.09
	4	56.49	51.30
	5	76.79	67.89
	**Mean**	64.64	58.81
SGD	1	20.78	7.17
	2	20.29	6.85
	3	23.21	8.75
	4	21.75	8.22
	5	20.94	9.35
	**Mean**	21.40	8.07
Nadam	1	81.66	81.30
	2	78.73	78.45
	3	78.73	78.69
	4	77.27	77.25
	5	79.22	79.14
	**Mean**	79.12	78.92

**Table 6:** Performance comparison of different deep learning models with proposed CNN optimized approaches.

Model	Accuracy (%)	Precision (%)	Recall (%)	F1 Score (%)
LeNet	39.5	44	37	39
AlexNet	24	3	12	5
Xception	47	64	54	43
Inception	69	76	61	62
ResNet	69	60	62	67
MobileNet	66	66	65	63
Proposed CNN Optimized with RMSprop (traditional training-validation splits)	89.09	88	85	86
Proposed CNN Optimized with Nadam (cross-validation)	79.12	79	78	78.92

## Data Availability

The tomato pest image dataset that supports the findings of this study is openly available in the https://data.mendeley.com/datasets/s62zm6djd2/1 (accessed on 15 February 2024).
